# Appended Aromatic Moieties in Flexible Bis‐3‐chloropiperidines Confer Tropism against Pancreatic Cancer Cells

**DOI:** 10.1002/cmdc.202000814

**Published:** 2020-12-03

**Authors:** Caterina Carraro, Tim Helbing, Alexander Francke, Ivonne Zuravka, Alice Sosic, Michele De Franco, Valentina Gandin, Barbara Gatto, D. Richard Göttlich

**Affiliations:** ^1^ Department of Pharmaceutical and Pharmacological Sciences University of Padova Via Francesco Marzolo 5 35131 Padova Italy; ^2^ Institute of Organic Chemistry Justus Liebig University Giessen Heinrich-Buff-Ring 17 35392 Giessen Germany

**Keywords:** anticancer agents, DNA alkylation, nitrogen mustards, pancreatic cancer, spheroids

## Abstract

Nitrogen mustards (NMs) are an old but still largely diffused class of anticancer drugs. However, spreading mechanisms of resistance undermine their efficacy and therapeutic applicability. To expand their antitumour value, we developed bis‐3‐chloropiperidines (B‐CePs), a new class of mustard‐based alkylating agent, and we recently reported the striking selectivity for BxPC‐3 pancreatic tumour cells of B‐CePs bearing aromatic moieties *embedded* in the linker. In this study, we demonstrate that such tropism is shared by bis‐3‐chloropiperidines bearing *appended* aromatic groups in flexible linkers, whereas esters substituted by aliphatic groups or by efficient DNA‐interacting groups are potent but nonselective cytotoxic agents. Besides, we describe how the critical balance between water stability and DNA reactivity can affect the properties of bis‐3‐chloropiperidines. Together, these findings support the exploitation of B‐CePs as potential antitumour clinical candidates.

## Graphical abstract

**Balancing act**: Following our development of bis‐3‐chloropiperidines (B‐CePs), a new class of sustainable mustard‐based alkylating agents, we demonstrate that B‐CePs bearing appended aromatic groups in linkers show tropism toward pancreatic tumour models. In addition, we describe how the critical balance between DNA reactivity and water stability can affect the properties of this class of compounds.

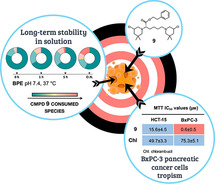

## Introduction

**Figure 1:**
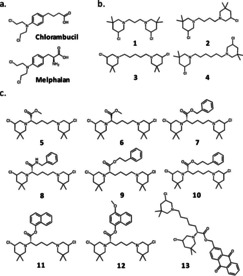
Chemical structures of a) chlorambucil and melphalan and of the analysed bis‐3‐chloropiperidines bearing b) linear alkyl and c) Lys ester/amide linkers.

Simplicity in synthesis coupled to the possibility of ample decoration of the molecular structure led us to build a new library of 3‐chloropiperidines as nucleic acids alkylating agents.^[1]^ Our project has been inspired by antitumour drugs with renowned therapeutic value such as chlorambucil and melphalan, old and inexpensive agents still widely used in clinic (Figure 1a).^[2]^ Nevertheless, widespread resistance phenomena and unwanted side effects undermined the efficacy of the oldest mustard‐based chemotherapies and new leads are urgently needed.^[3]^ In this direction, we demonstrated that bifunctional 3‐chloropiperidines (B‐CePs) do react with guanine nucleobases as intended, thus inducing damage on isolated as well as genomic DNA.[^1d^, ^1f^] Analysing our library on model DNA, we proved that flexible aliphatic linkers connecting the two reactive centres of B‐CePs (Figure 1b) confer high alkylating potencies in vitro, whereas restrained flexibility of the linker decreases the reactivity of the analogues.^[1a]^ Nonetheless, compounds with lower reactivity showed to be quite cytotoxic in tumour cells, making them the best suited candidates for further development.^[1f]^ Strikingly, B‐CePs bearing linkers with *embedded* aromatic groups disclosed an unexpected tropism against a pancreatic cancer cell line (BxPC‐3) poorly sensitive to chlorambucil.^[1f]^ Accordingly, derivative **3** bearing a linear pentane linker (Figure 1b) lacked the preferential activity exerted by aromatic analogues against pancreatic cancer cells.^[1f]^

Following this line of evidence, we investigate here whether aromatic moieties *appended* to the flexible aliphatic side chain of **3** could reproduce the cytotoxicity and pancreatic tumour tropism evidenced for rigid aromatic B‐CePs.^[1f]^ To do that, we expanded our library of compounds bearing lysine linkers variously derivatized on the carboxyl group,[^1b^, ^1c^] and completed the evaluation of their cytotoxicity and reactivity toward the biologically relevant nucleophiles.

In detail, the l‐Lys ester B‐CePs set analysed here included (Figure 1c): i) compound **5** bearing a methyl ester function and the aromatic derivatives **7**, **9**, **10** with increasing distance between the phenyl ring and the carbonyl centre;^[1b]^ ii) the naphthyl‐substituted compounds **11** and its *para*‐methoxylated analogue **12**;^[1b]^ iii) the anthraquinone (AQ) conjugate **13**, whose ability of binding DNA noncovalently has been already highlighted.^[1c]^ Along with the previously described compounds, the new derivative **6**, the unnatural d‐Lys methyl ester enantiomer of **5**, was synthesised and tested to assess the possible influence of stereogenicity on the activity of Lys ester derivatives. In addition, compound **8** bearing a l‐Lys amide linker was synthesised and compared to its ester analogue **7**. Besides, to assess the effect of length in *linear alkyl* linkers on the cytotoxicity of derivatives we also tested compounds **1**, **2** and **4** (Figure 1b).^[1a]^

Results confirmed that *appended* aromatic moieties in the lysine linker confer to B‐CePs preferential activity against the BxPC‐3 pancreatic cell line observed for analogues bearing *embedded* aromatic groups.^[1f]^ The test compounds showed valuable indexes of cytotoxicity in 2D and 3D BxPC‐3 cell cultures, linked to different consumption kinetics in aqueous buffer, long‐term reactivity with DNA and efficient cellular uptake mediated by transporters. From these premises, this study completes the investigation on the reactivity of lysine esters while answering the question of the chemical determinants for the tropism of B‐CePs against BxPC‐3 cells, further directing the development of this class of alkylators as anticancer agents.

## Results and Discussion

**Scheme 1:**
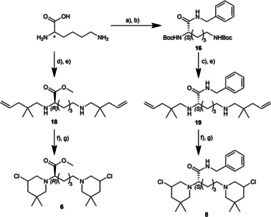
Synthesis of new bis‐3‐chloropiperidine derivatives **6** and **8**. a) Boc_2_O, 1 M NaOH, H_2_O/dioxane (1 : 1), RT, 12 h; b) ClCO_2_Et, NEt_3_, benzyl amine, dioxane, RT, 12 h; c) TFA, CH_2_Cl_2_, 0 °C to RT, 3 h; d) 2,2‐dimethoxypropane, conc. HCl, MeOH, reflux to RT, 15 h; e) 2,2‐dimethylpent‐4‐enal,^[5]^ NaBH(OAc)_3_, AcOH, dry CH_2_Cl_2_, 0 °C to RT, 12 h; f) NCS, dry CH_2_Cl_2_, 0 °C to RT, 2.5–3.5 h; g) cat. TBAI, dry CHCl_3_, 60 °C (oil bath temperature), 2 h, (inseparable diastereomeric mixture).

The synthesis of bis‐3‐chloropiperidines **1**–**4** as well as **5**, **7**, **9**–**12** and **13** has already been described elsewhere.[^1a^, ^1b^, ^1c^] The new B‐CeP derivatives **6** and **8** were synthesised using a similar strategy (Scheme 1). To apply our well‐established procedure of N‐chlorination and iodine catalysed cyclization^[4]^ the unsaturated diamines **18** and **19** had to be synthesised. For the amide analogue of compound **7** both free amino groups of l‐Lysine where protected using di‐*tert*‐butyl dicarbonate (Boc_2_O), in order

to achieve a clean amide coupling with benzyl amine. This coupling reaction was accomplished by activation of the free carboxyl group with ethyl chloroformate and provided the Boc‐protected benzylamide derivate **16** in very good yield. After deprotection with trifluoroacetic acid the resulting free diamine was converted to the desired unsaturated diamine **19** by double reductive amination with 2,2‐dimethylpent‐4‐enal.^[5]^

In contrast, the synthesis of compound **6**, the enantiomer of B‐CeP **5**, was carried out without any protecting groups. The starting material, in this case d‐Lysine, was directly converted into its corresponding methyl ester hydrochloride salt by treatment with 2,2‐dimethyoxypropane and hydrochloric acid. After another double reductive amination with 2,2‐dimethylpent‐4‐enal the unsaturated diamine **18** was obtained. Both secondary diamines **18** and **19** where then reacted with *N*‐chlorosuccinimide and the resulting bis*‐N‐*chloroamines converted to their corresponding bis*‐*3‐chloropiperidines **6** and **8** by iodine catalysed cyclization.

### Appended aromatic groups in B‐CePs confer tropism against BxPC‐3 pancreatic cancer cells

To further characterize the antiproliferative properties of B‐CePs and to gain new chemical insights on the mentioned tropism for pancreatic cancer cells, we investigated the cytotoxicity of compounds in Figure 1b and 1c against HCT‐15 (colorectal adenocarcinoma), 2008 (ovarian carcinoma) and BxPC‐3 (pancreatic adenocarcinoma) tumour cell lines. Table 1 reports the MTT assay IC_50_ values with associated standard deviations obtained after 72 h of incubation of cells with test compounds.^[6]^ Values for compound **3** and the reference nitrogen mustard chlorambucil were published in our previous works.[^1e^, ^1f^]

**Table 1:** MTT assay IC_50_ values with associated SDs of test bis‐3‐chloropiperidines against HCT‐15, 2008 and BxPC‐3 cells after 72 h of treatment. IC_50_ values were calculated from two independent experiments by a four‐parameter logistic model. Values highlighted in red: IC_50_≤1.0 μm; in yellow: 1.1≤IC_50_≤6.0 μm; in green: 6.1≤IC_50_≤15.0 μm; in blue: IC_50_≥15.1 μm.

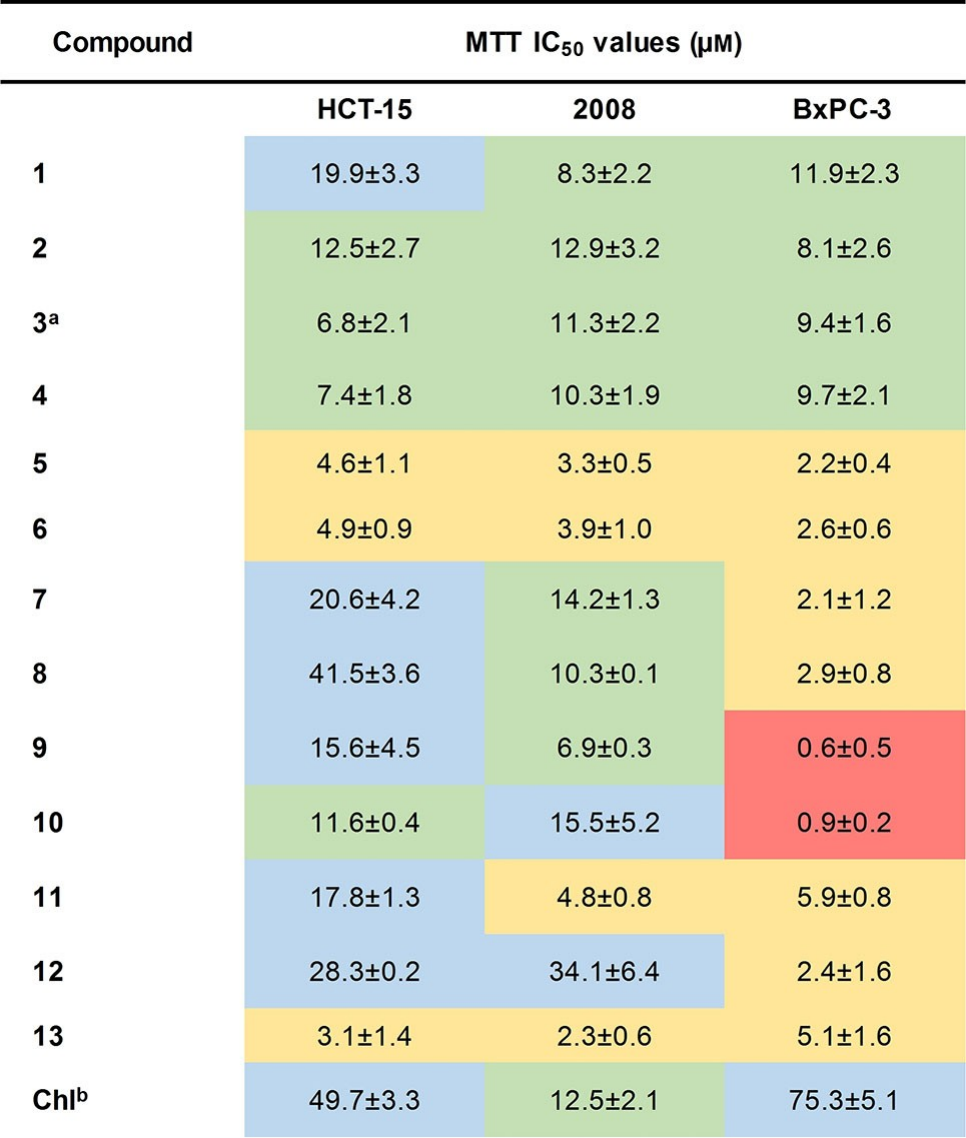

[a] MTT IC_50_ from our previous work^[1f]^ [b] Chlorambucil MTT IC_50_ from our previous work[^1e^, ^1f^]

Noteworthy, all test B‐CePs were more active than chlorambucil against HCT‐15 and BxPC‐3 cells, and most of them also against 2008 cells, confirming the promising potential for this class of compounds to improve the therapeutic profile of NMs. Clearly, no tropism toward pancreatic cancer cells was observed for B‐CePs with linear alkyl linkers (**1**–**4**), in line with our previous observation.^[1f]^ While MTT IC_50_ values of **1**–**4** were similar and attested around 10 μm in 2008 and BxPC‐3 cells, the longer alkyl chains of **3** and **4** improved the cytotoxic effect in HCT‐15 cells.

The introduction of the methyl ester group in **5** and **6** increased the cytotoxicity in comparison with their alkyl analogue **3**, regardless of the stereochemistry of the Lys bridging linker. The high reactivity of the unsubstituted linear alkyl B‐CeP **3**, previously observed in plasmid DNA cleavage assays,^[1b]^ turned out to be detrimental in cells, possibly accelerating the premature reaction with competing nucleophiles in the cellular milieu. Consistently, given their reported low potency toward isolated DNA,^[1b]^ B‐CePs Lys ester substituted with aromatic moieties (**7**–**12**) showed to be quite effective against tested tumour cells. The substitution with *appended* aromatic groups hallmark the higher toxicity against the pancreatic tumour cell line, in line with the preferential activity against BxPC‐3 cells reported for B‐CePs with *embedded* aromatic linkers.^[1f]^ Noticeably, the AQ conjugate **13** was rather cytotoxic but not cell‐line‐selective compared to **7**–**12**, a feature most likely related to its peculiar reactivity empowered by efficient DNA intercalating properties.^[1c]^ In the ester series **7**, **9** and **10**, the shorter side chain of **7** appeared to be less suited in cells, despite the earlier observation that **7** and **9** show similar reactivity with plasmid DNA.^[1b]^ Indeed, the aromatic compounds **9** and **10** achieved nanomolar IC_50_s against pancreatic cancer cells (BxPC‐3 IC_50_ **9**: 0.6±0.5 μm; BxPC‐3 IC_50_ **10**: 0.9±0.2 μm). Concerning the newly synthesised derivatives, we observed a similar profile between the Lys ester **7** and its amide analogue **8**.

In light of the promising activity of **9** and **10** on monolayer cultures of BxPC‐3 cells, the two compounds were tested against spheroids of the same cancer cell line. Three‐dimensional cultures better simulate the complexity of solid tumours, both in terms of cellular heterogeneity among concentric spheroid layers and approximation of the microenvironmental cell‐extracellular matrix (ECM) interactions.^[7]^ Cell viability in spheroids exposed to compounds **9** and **10** for 72 h was assessed by the acidic phosphatase (APH) assay and IC_50_s with associated standard deviations are reported in Table 2.^[8]^

**Table 2:** APH assay IC_50_ values with associated SDs against 3D BxPC‐3 cultures after 72 h of treatment. IC_50_ values of compounds **9** and **10** were calculated from two independent experiments by a four‐parameter logistic model.

Compound	APH IC_50_ values [μm]
**9**	71.8±1.9
**10**	105.6±0.9
Chl	inactive

Chl: chlorambucil, inactive at the maximum tested concentration (200 μm).

Compounds **9** and **10** demonstrated to be active also against BxPC‐3 spheroids, an encouraging result considering the inactivity of chlorambucil up to the maximum tested concentration of 200 μm. As observed in monolayer cultures, **9** was more active than **10**, suggesting that the interplay between compounds reactivity and lipophilicity are important factors to be considered in spheroid models.

### Aromatic moieties reduce B‐CePs hydroxylation

**Figure 2:**
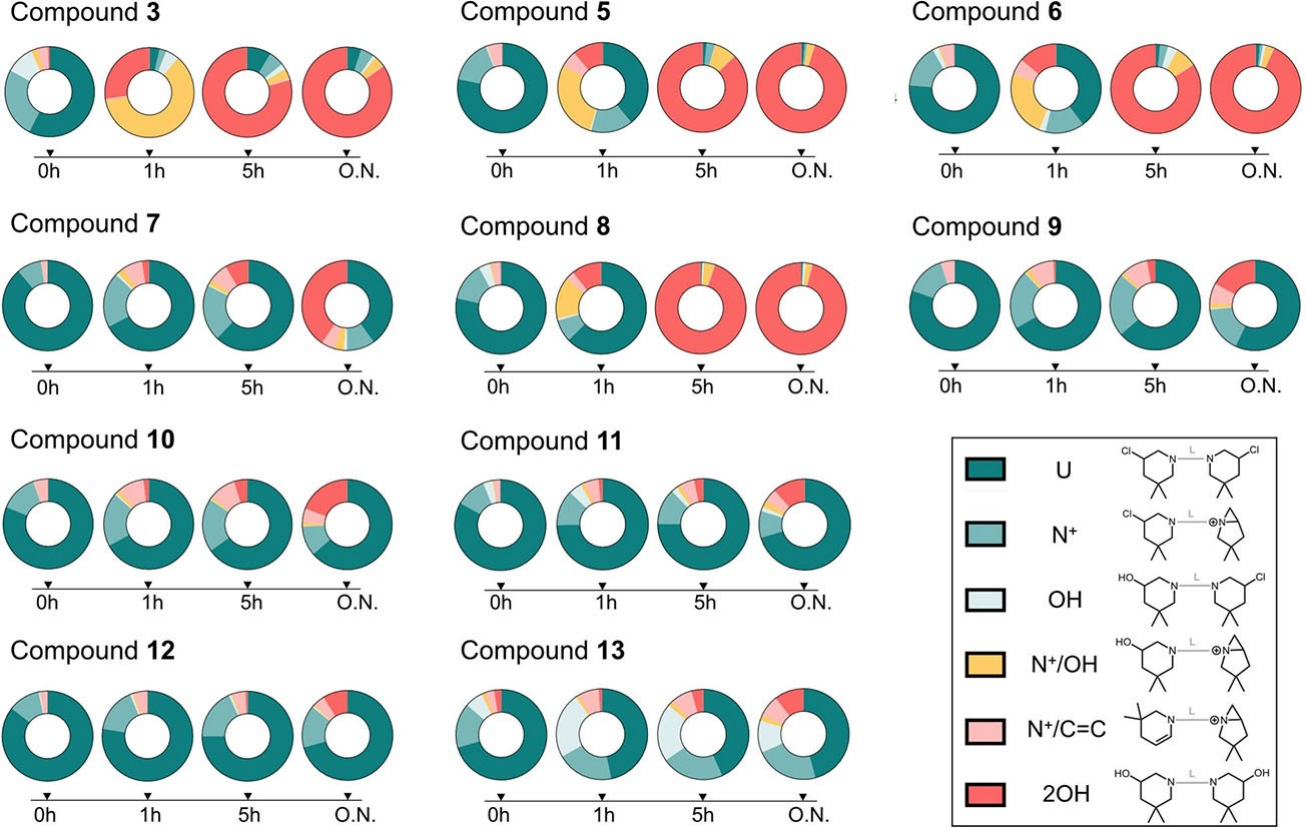
Hydroxylation of B‐CePs. Time‐followed formation of B‐CePs reaction species at 37 °C in BPE buffer pH 7.4 as detected by ESI‐MS. Graphs report the relative percentages of reaction species (whose symbols and structures are schematized in the legend) resulting from the incubation test B‐CePs in BPE buffer pH 7.4 at 37 °C for 0, 1, 5 h and overnight (O.N.) detected by ESI‐MS.

The highly electrophilic nature of B‐CePs accounts for their ability to react with nucleophiles, such as water molecules and nucleobases in DNA.[^1d^, ^9^] In aqueous buffers, 3‐chloropiperidines spontaneously rearrange into the aziridinium ion (N^+^), which is readily attacked by water giving rise to mono‐ (OH) and dihydroxylated (2 OH) products, irreversibly.[^1b^, ^1d^] Since the side‐reaction with water abates the fraction of compound still able to alkylate the biological target, the rate of hydroxylation of 3‐chloropiperidine rings is informative both of B‐CePs reactivity and of their consumption kinetics. We therefore investigated by ESI‐MS the hydroxylation of B‐CePs **5**–**13**. ESI‐MS experiments with water were performed in BPE pH 7.4, the buffer employed to assay compounds reactivity with isolated DNA. Freshly‐made solutions of compounds in buffer were analysed immediately or after 1 h, 5 h and overnight (O.N.) incubation at 37 °C by ESI‐MS using the protocol reported in previous studies..[^1a^, ^1b^, ^1c^, ^1e^, ^1f^] Graphs in Figure 2 show the relative percentage of B‐CePs reaction species, whose chemical structures are schematized in the figure legend. Graph‐associated relative percentages are reported in Table S1 in the Supporting Information.

As attested by results in Figure 2, the hydroxylation of all Lys derivatives generally slowed down in buffer at physiological pH compared to the aliphatic control **3** bearing an unsubstituted pentane linker. In agreement with their comparable cellular activity, the l‐Lys derivative **5** and its unnatural d‐Lys enantiomer **6** showed a very similar hydroxylation pattern in the buffered solution at physiological pH.

The amide **8** exhibited different reactivity profiles than the ester **7**: **8** underwent a faster hydrolysis compared to its ester analogue **7**, showing to be almost totally dihydroxylated after 5 h of incubation. Most likely the hydroxylation of **8** is accelerated by hydrogen bonding interactions of the amide NH, which is not possible for the ester analogue **7**. Indeed, all aromatic ester derivatives demonstrated to be mostly unreacted even after a prolonged overnight incubation in buffer. The slower consumption kinetics of aromatic ester derivatives **7**, **9**, **10**, **11** and **12** is consistent with their reported limited reactivity with purified DNA,^[1b]^ which might enable them to “preserve” their reactivity in the cell cytoplasm context, resulting in the considerable cytotoxicity shown above. Interestingly, **9** preserved its reactivity far longer than **7**: the fraction of dihydroxylated **9**_2OH_ is much lower compared to **7**_2OH_ after O.N. incubation. This feature may account for the higher cytotoxicity of **9** compared to **7**.

Notably, the AQ conjugate **13**, which was demonstrated to promote a considerably higher plasmid cleavage compared to the other aromatic Lys ester B‐CePs,^[1c]^ showed here a reduced hydrolysis pattern. This result further endorses the hypothesis that the improved DNA cleavage activity of **13** might depend on the optimized targeting of DNA mediated by the intercalating AQ moiety rather than simply a higher electrophilicity.^[1c]^

### Appended linkers modulate B‐CePs reactivity with DNA

**Figure 3:**
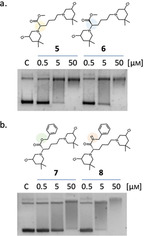
DNA cleavage activity of bis‐3‐chloropiperidines a) **5** versus **6** and b) **7** versus **8**. The supercoiled pBR322 plasmid was incubated with increasing concentrations of test compounds at 37 °C for 3 h in BPE buffer. Lower band: supercoiled plasmid form, upper bands: open circular plasmid species. C: supercoiled pBR322 plasmid control.

In previous studies, we demonstrated the ability of B‐CePs to cleave isolated DNA with potencies dependent on the chemical structure of the linker.[^1a^, ^1b^, ^1c^, ^1f^] Specifically, the insertion of a methyl ester function in **3** only slightly affected the DNA alkylation of B‐CeP **5**, while the presence of appended aromatic groups strongly curtailed the reactivity of compounds with DNA. This was particularly evident when increasing the length of the alkyl spacer between the ester function and the aromatic ring in **7**, **9** and **10**.^[1b]^ To complete the in vitro characterization of synthesised B‐CePs and to explore the suitability of appended linkers to modulate B‐CePs properties, we investigated the new derivatives **6** and **8** at the DNA cleavage assay. To appreciate an immediate comparison with their published analogues under the same experimental conditions,^[1b]^ the new compound **6** bearing the d‐Lys linker was tested along with its enantiomer **5**, while the new amide derivative **8** was tested along with the ester analogue **7**. The supercoiled pBR322 plasmid was incubated with increasing concentrations (0.5, 5, 50 μm) of test compounds for 3 h at 37 °C in BPE buffer pH 7.4, as reported previously.[^1a^, ^1b^, ^1c^, ^1e^, ^1f^] The ability of B‐CePs to cut DNA leads to a relaxation of the plasmid to its open circular form, visualized on 1 % agarose as a distinct upper band with lower electrophoretic mobility compared to the supercoiled parent. Results after 3 h of incubation are shown in Figure 3a (**5** vs. **6**) and b (**7** vs. **8**).

The d configuration of Lys linker of **6** seems not to affect its remarkable DNA cleavage activity compared to natural l‐Lys analogue **5**, a behaviour consistent with the similar reactivity in buffer and the observed cellular effects. On the other hand, the amide bond in the aromatic Lys linker of **8** substantially increased its DNA cleavage potency compare to the less potent ester analogue **7**, presumably again due to possible hydrogen bonding interactions. In both cases, results are in line with the reactivity observed in BPE buffer at the ESI‐MS. In Figure 3b, we observe slower DNA migration at 50 μM compound, possibility hinting to noncovalent binding by **7** and **8** to the nucleic acid.

### Water‐stable B‐CePs retain long‐term DNA cleavage properties

Having demonstrated that aromatic ester and amide B‐CePs do cleave DNA and in light of the new findings on their reduced hydroxylation in buffered conditions, we checked if reactivity with DNA is retained even after a prolonged incubation in aqueous buffer. To do this, we compared the amide **8** with the esters **7** and **9**: as shown in Figure 2, they have quite different hydroxylation kinetics in buffer, with **8** being faster consumed in BPE than **7** and **9**.

**Figure 4:**
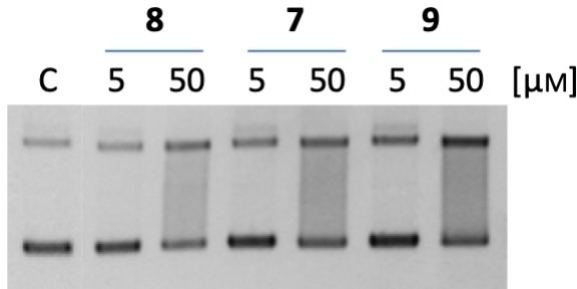
DNA cleavage activity of bis‐3‐chloropiperidines **8**–**9** after overnight preincubation of compounds at 37 °C in BPE buffer (pH 7.4). Preincubated compounds were added at increasing concentrations to the pBR322 plasmid, and the mixture was incubated at 37 °C for 3 h. C: supercoiled pBR322 plasmid control.

The results of the plasmid cleavage assay by this set of compounds after a prolonged DNA‐free preincubation in buffer at 37 °C is reported in Figure 4.

As expected, the DNA cleavage was clearly reduced by the overnight preincubation in aqueous buffer, as the sustained hydrolysis abates the fraction of compound still able to react with the plasmid. However, **7** and **9** preserved higher long‐term DNA cleavage ability, as lower was their water consumption detected by ESI‐MS relative to **8** (Figure 2). The balance between hydroxylation rate and reactivity with DNA is one of the key elements to determine the biological value of these compounds: if slowly consumed by competing nucleophiles, B‐CePs preserve the ability to react with their intended nuclear target.

### Appended aromatic moieties in B‐CePs promote noncovalent DNA interactions

**Figure 5:**
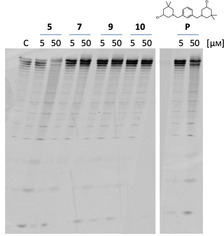
Denaturing polyacrylamide gel showing concentration‐dependent cleavage of a 22‐mer double‐stranded oligonucleotide caused by bis‐3‐chloropiperidine alkylation of guanines. The 5’‐FAM‐labelled scrambled duplex oligonucleotide (4 μm), GGA TGT GAG TGT GAG TGT GAG G, was treated with indicated compounds at 37 °C in BPE buffer pH 7.4 at 5 and 50 μm for 24 h. Products were run on denaturing polyacrylamide gel 20 % in TBE buffer. C: untreated duplex oligonucleotide control.

The retarded open circular plasmid form visualized in Figure 3b suggested possible noncovalent interactions between aromatic B‐CePs and DNA, as already proved for the naphthyl‐substituted derivatives **11** and **12** and, more evidently, for the AQ conjugate **13**.[^1b^, ^1c^] To verify noncovalent DNA‐interacting properties of *appended* mono‐cyclic aromatic B‐CePs, we incubated the lysine esters **7**, **9** and **10** with a double‐strand G‐rich oligonucleotide for 24 h at 37 °C, and then observed results on a denaturing polyacrylamide gel (Figure 5). For comparative purposes, we analysed also the recently published derivative bearing an *embedded* aromatic linker (**P**, whose chemical structure is reported in Figure 5),^[1f]^ and the aliphatic ester **5**.

Only B‐CePs bearing aromatic moieties demonstrated to interact noncovalently with the oligonucleotide, as attested by the appearance of a weak but distinctive uppermost band with retarded gel migration. This band, not detected upon incubation with **5**, was more pronounced in the case of the compounds bearing *appended* phenyl‐methyl and phenyl‐ethyl substituent (**7** and **9**), while minor for the B‐CePs **P** bearing an *embedded* aromatic linker, suggesting that only sufficiently spaced and unhindered aromatic rings can establish stacking interactions with DNA in B‐CePs series. The higher reactivity of **5** with DNA is re‐attested here by the concentration dependent decrease of the intact oligo intensity and concurrent increased intensity of cleaved oligo bands.

### Transporters mediate entry of Lys ester B‐CePs in BxPC‐3 cells

**Figure 6:**
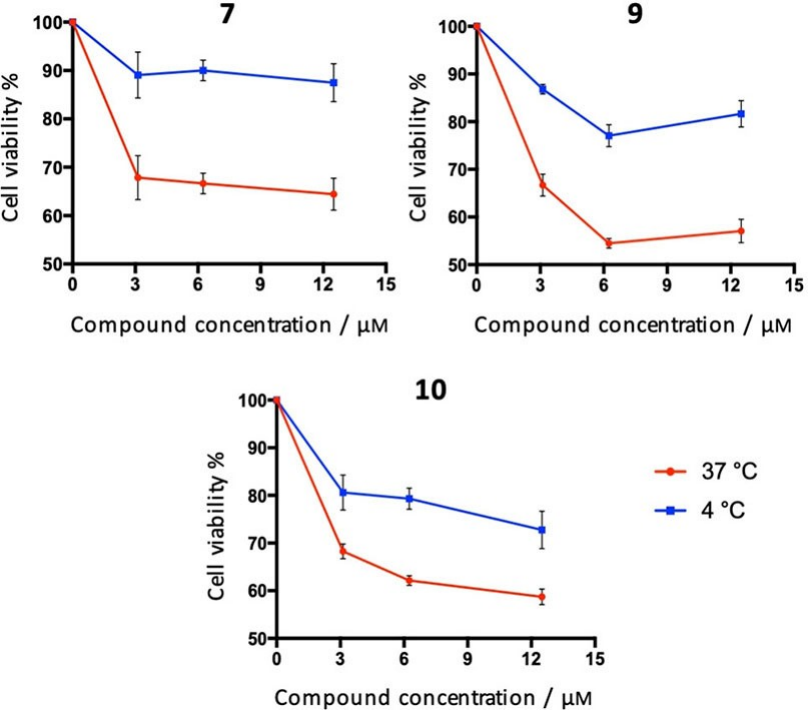
BxPC‐3 cell viability upon exposure to increasing concentrations of selected compounds **7**, **9** and **10** for 5 h at 37 or 4 °C, followed by a gentle rinse of wells with PBS and addition of fresh RPMI medium. The MTT assay was performed after 72 h. Average viability percentages with associated SDs from three experimental replicates are reported.

B‐CePs were recently demonstrated to exploit mechanisms of transporter‐mediated uptake in BxPC‐3 cells.^[1f]^ In order to confirm such mode of entry for the new set of Lys linker derivatives, a modified MTT assay was performed on the highly active compounds **7**, **9** and **10**.[^1f^, ^10^] As reported in previous works, cells were seeded in two microplates and incubated in parallel at 37 °C or 4 °C for 5 h.^[1f]^ Afterwards, fresh medium was added to rinsed wells, microplates were further incubated at 37 °C and cell viability was assessed at 72 h. Results are reported in Figure 6.

Given the almost identical values of formazan absorbance between untreated controls in plates incubated at 37 and 4 °C, we excluded that differences in viability might depend on the sole initial temperature gap. At 37 °C (red line), the intracellular accumulation of compounds takes advantage of both passive diffusion and transporter‐mediated uptake. Conversely, since transporters have been shown to be inhibited at low temperatures, the cytotoxicity observed at 4 °C (blue line) depends almost exclusively on the passive diffusion of compounds.^[10]^ Thus, the elative contribution of transporters to the intracellular accumulation of B‐CePs is described by the difference in viability resulting from the 5 h parallel incubation of microplates at 37 °C or 4 °C. On these premises, graphs in Figure 6 confirm that also appended aromatic Lys ester derivatives exploit transporters to re‐enter BxPC‐3 cells. The contribution of transporters to B‐CePs

intracellular accumulation is comparable between **7** and **9**, while the role of transporters in the uptake of **10** is less relevant. In fact, most likely for the higher lipophilicity conferred by its longer benzyl side chain, **10** takes advantage of an enhanced passive diffusion, a slower and less efficient process. This feature could partly explain the higher cytotoxicity of **9** compared to **10** especially against spheroids, where an efficient transporter‐mediated compound distribution across inner layers is critical.

## Conclusion

B‐CePs are a class of DNA alkylating agents developed to overcome the limitations of available chemotherapeutics.[^1a^, ^1b^, ^1c^, ^1d^, ^1f^] To this extent, we recently reported the promising anticancer properties of a set of B‐CePs with *embedded* aromatic linkers and their notable tropism against BxPC‐3 pancreatic tumour cells.^[1f]^ As a step forward, in this study we analysed Lys esters along with an amide analogue to further characterize their mechanism of action and establish the tropism against BxPC‐3 cells.

By virtue of their electrophilic nature, B‐CePs react with nucleophiles, such as water and DNA nucleobases:[^1a^, ^1d^] the balance between nucleic acids alkylation and hydroxylation of 3‐chloropiperidine rings contributes to delineate the pharmacological activity of these compounds. The cellular evaluation of B‐CePs demonstrated their generally improved potency compared to the therapeutic NM chlorambucil against the tested colorectal, ovarian and pancreatic cancer cell lines. In detail, we observed that the Lys methyl ester linker increased the cytotoxicity in all cell lines compared to the linear alkyl analogue, whose sustained hydroxylation leads to premature consumption in aqueous environment. In this sense, the limited reactivity with water implies a much slower consumption of compounds in buffered conditions and the retention of long‐term reactivity with DNA.

Notably, we point out in this study the remarkable activity in pancreatic cancer cells by all B‐CePs substituted with *appended* aromatic linkers: the aromatic lysine esters **9** and **10** resulted the most active among the test derivatives, exhibiting nanomolar IC_50_ values against the pancreatic cancer cell line. It is significant that these two derivatives were demonstrated to be active against the 3D model of BxPC‐3 spheroids, possibly thanks to efficient cell accumulation. Aromatic moieties *appended* to the flexible linkers can therefore reproduce the pancreatic tumour tropism and cytotoxicity evidenced for rigid *embedded* aromatic B‐CePs,^[1f]^ suggesting the existence of collateral mechanisms of toxicity along with DNA alkylation and damage. In fact, the efficiency of DNA repair machineries, drug permeability and efflux mechanisms as well as the metabolic status of cells are known to determine distinct sensitivities to treatments in different cancer types, even in the case of drugs exerting non‐targeted mechanism of action, such as NMs.^[11]^ Transcriptome profiling to identify cancer vulnerabilities is gaining increasing relevance and applicability in drug discovery.^[12]^ Studies on the dynamics of the transcriptional and chromatin states in cells exposed to these cytotoxic compounds are ongoing to mine the complex basis of the tropism of aromatic B‐CePs for the pancreatic cancer cell line.

## Experimental Section

### Materials and methods

**MS studies**: B‐CePs stability and hydrolysis rate upon incubation at 37 °C in BPE (NaH_2_PO_4_ 0.2 mm, Na_2_HPO_4_ 0.6 mm, Na_2_EDTA 0.1 mm) pH 7.4 was followed in time by ESI‐MS. A fresh solution of test compounds at the concentration of 80 μm was prepared in BPE buffer from 1 mm DMSO stocks and incubated at 37 °C. Samples were taken after 0 h, 1 h, 5 h and overnight incubation, diluted 1 : 10 with methanol and analysed by ESI‐MS. Measurements were performed in positive ion mode using a Xevo G2‐XS Qtof instrument (Waters) and calculations were performed using the MassLynx software (Waters). The relative abundances of detected reaction species at different experimental conditions were reported as percentages.[^1f^, ^13^]

**Cleavage assay**: The DNA cleavage properties of test B‐CePs were assessed on the supercoiled plasmid pBR322 (Inspiralis Ltd). The cleavage assay was performed following previously reported materials and protocols.[^1b^, ^1d^, ^1e^] B‐CePs dilutions were freshly prepared from a 10 mm DMSO stock in Milli‐Q water.

**Sequencing gel analysis**: The sequencing gel analysis was performed according to previously reported materials and protocols.[^1a^, ^1c^, ^1d^] The 5’‐FAM‐labelled scrambled oligonucleotide (5’‐FAM‐GGA TGT GAG TGT GAG TGT GAG G‐3’, Eurogentec) was slowly annealed with its complementary co‐scrambled oligonucleotide (5’‐CCT CAC ACT CAC ACT CAC ATC C‐3’, Eurogentec): equimolar amounts of both sequences were mixed in BPE buffer, denatured at 95 °C for 5 min and left to cool to room temperature to obtain the final duplex. The 5’‐FAM‐labelled duplex oligonucleotide (4 μm) was incubated with 50 μm of test derivatives in BPE buffer for 24 h at 37 °C. Samples were dried in a vacuum centrifuge (UNIVAPO 100H, UniEquip), resuspended in denaturing gel loading buffer (10 mm Tris‐HCl, 80 % formamide, 0.025 % bromophenol blue), and loaded on a 20 % denaturing polyacrylamide gel (7 m urea) in TBE buffer. The fluorescence of the oligonucleotide bands was detected by scanning using Storm Scanner Control (STORM 840, Molecular Dynamics).

**Cell cultures**: Colon (HCT‐15) and pancreatic (BxPC‐3) carcinoma cell lines were purchased from ATCC (American Type Culture Collection) while the human ovarian 2008 cancer cell line was provided by G. Marverti (Department of Biomedical Science, University of Modena and Reggio Emilia, Modena, Italy). Cell lines were maintained in logarithmic phase at 37 °C under 5 % CO_2_ using RPMI‐1640 medium (Euroclone) containing 10 % foetal calf serum (FCS, Euroclone), antibiotics (50 units/mL penicillin and 50 μg/mL streptomycin), and 2 mm l‐glutamine.

**MTT assay**: The MTT assay was performed as in previously reported materials and protocols.[^1e^, ^1f^, ^6^] Test compounds were dissolved in DMSO and added at defined concentrations to the cell growth medium to a final solvent concentration of 0.5 %, which had no detectable effect on cell killing. Compounds MTT IC_50_s were extrapolated applying a four‐parameter logistic (4‐PL) model and average values with associated SDs of two independent experiments (each one with three replicates) were reported. We applied modifications to the classical MTT assay to investigate the uptake of B‐CePs. BxPC‐3 cells were incubated with test compounds for 5 h, either at 37 or 4 °C in parallel microplates. Then, wells were rinsed with PBS, fresh RPMI was added and microplates were incubated at 37 °C in the absence of compound. After 72 h from seeding, cell viability was assessed and average values with associated SDs from three experimental replicates were reported.[^1f^, ^10^]

**Acid phosphatase (APH) assay**: The APH assay was performed to assess cell viability of 3D BxPC‐3 cultures treated with test B‐CePs following previously reported protocols.[^1f^, ^8^] Cells were seeded (1.5×10^3^ cells/well) in phenol red‐free RPMI medium (Euroclone) containing antibiotics and l‐glutamine as well as 10 % FCS and 20 % methyl cellulose in round‐bottom non‐tissue culture treated 96 well‐plates (Greiner Bio‐one). After 72 h from seeding, spheroids were incubated with defined concentration of compound freshly dissolved in DMSO to a final solvent concentration of 0.5 %. After 72 h of treatment, spheroids were incubated with 100 μL of the assay buffer for 3 h at 37 °C (0.1 m sodium acetate, 0.1 % Triton‐X‐100, supplemented with ImmunoPure *p*‐nitrophenyl phosphate by Sigma). Subsequently, 10 μL of 1 m NaOH were added before measuring the absorbance of each well at 405 nm (BioRad 680 microplate reader). Compounds IC_50_s were extrapolated applying a four‐parameter logistic (4‐PL) model and average values with associated SDs of two independent experiments (each one with three replicates) were reported.

### Chemistry

All solvents were purified by distillation prior to use and in case of anhydrous solvents dried and stored under nitrogen atmosphere. Commercially available reagents were used as supplied if not stated different. Synthesis using anhydrous solvents were carried out under Schlenk conditions. For purification by flash chromatography silica gel 60 (Merck) was used. ^1^H and ^13^C NMR spectra were recorded at Bruker Avance II 200 spectrometer (^1^H at 200 MHz; ^13^C at 50 MHz) and Bruker Avance II 400 spectrometer (^1^H at 400 MHz; ^13^C at 100 MHz) in deuterated solvents. Chemical shifts were determined by reference to the residual solvent signals. High‐resolution ESI mass spectra (HRMS) were recorded in methanol using a ESImicroTOF spectrometer (Bruker Daltonics) in positive ion mode. The synthesis and analytical data of the bis‐3‐chloropiperidines **1**–**5, 7, 9**–**12** ^[1b]^ and **13** ^[1c]^ have been described elsewhere. Copies of the ^1^H and ^13^C NMR spectra as well as the HRMS spectra of the new B‐CeP derivatives **6** and **8** are included in the supporting information.

### Synthetic procedures

**d‐Lysine methylester dihydrochloride (14)**: To a suspension of d‐lysine hydrochloride (5.00 g, 22.82 mmol) in methanol (55 mL) was added 2,2‐dimethoxypropane (35 ml) and conc. HCl (9 mL). The mixture was heated to reflux for 3 h and was then stirred at room temperature for 12 h. Afterwards the solvent was removed under reduced pressure. The residue was dissolved in a small amount of methanol and TBME (250 mL) was added at 0 °C to induce crystallization. The crude product was then recrystallized from TBME/methanol to obtain the title compound (**14**) as a white solid (5.05 g, 21.68 mmol, 95 %). ^1^H NMR (400.1 MHz, D_2_O): *δ*=4.20 (t, *J*=6.5 Hz, 1H), 3.87 (s, 3H), 3.04 (t, *J*=7.7 Hz, 2H), 2.12–1.91 (m, 2H), 1.75 (p, *J*=7.7 Hz, 2H), 1.62–1.42 (m, 2H) ppm; ^13^C NMR (100.6 MHz, D_2_O): *δ*=170.6, 53.6, 52.6, 39.0, 29.2, 26.2, 21.5 ppm; HRMS(ESI): *m/z* calcd for C_7_H_17_N_2_O_2_: 161.1285, found 161.1297 [*M*+H^+^].

**(2*S*)‐2,6‐Bis(*tert*‐butoxycarbonylamino)lysine (15)**: To a solution of l‐lysine monohydrate (5.09 g, 31.0 mmol) in water/dioxane (1 : 1, 100 mL) were added di‐*tert*‐butyl dicarbonate (16.9 g, 78.0 mmol) and 1 M NaOH_(aq)_ (35 mL). The reaction mixture was stirred at room temperature for 12 h and then concentrated in vacuo until approximately 50 mL remained. The pH was adjusted to 1–2 by careful addition of an aqueous KHSO_4_ solution (150 g/L). The suspension was extracted three times with ethyl acetate. The combined organic layers were dried over MgSO_4_ and the solvent was removed under reduced pressure to afford the title compound (**15**; 9.89 g, 28.5 mmol, 92 %) as a colourless oil. ^1^H NMR ([D_6_]DMSO, 200.1 MHz): *δ*=12.40 (s, 1H, OH), 6.98 (d, J=7.9 Hz, 1H), 6.76 (t, J=5.4 Hz, 1H), 3.75–3.86 (m, 1H), 2.50–2.83 (m, 2H), 1.37 (s, 18H), 1.14–1.68 (m, 6H) ppm; ^13^C NMR ([D_6_]DMSO, 50.3 MHz): *δ*=173, 155.1, 77.4, 76.8, 52.9, 29.9, 28.6, 27.7, 22.4 ppm (one signal, probably around 40 ppm, is hidden by the solvent signal). HRMS(ESI): *m/z* calcd for C_16_H_30_N_2_O_6_Na^+^: 369.1996, found 369.1998 [*M*+Na^+^].

**(2*S*)‐*N*‐Benzyl‐2,6‐bis(*tert*‐butoxycarbonylamino)hexanamide (16)**: To a solution of (2S)‐2,6‐bis(*tert*‐butoxycarbonylamino)lysine (**15**; 1.00 g, 2.89 mmol) in 1,4‐dioxane (20 mL) triethylamine (0.35 g, 3.47 mmol) as well as ethyl chloroformate (0.38 g, 3.47 mmol) were added. The solution was stirred at room temperature for 20 min. Afterwards benzylamine (0.37 g, 3.47 mmol) was added dropwise, and the mixture was stirred at room temperature for 12 h. The solvent was removed under reduced pressure and the residue dissolved in ethyl acetate (30 mL). The solution was washed with distilled water, followed by 1 M NaOH_(aq)_, 1 M HCl_(aq)_ and distilled water. The organic phase was separated, dried over MgSO_4_ and the solvent was removed under reduced pressure. The crude product was purified by flash column chromatography (pentane/ethyl acetate 1 : 1) to obtain the title compound (**16**) as a colourless oil (1.16 g, 2.66 mmol, 92 %). ^1^H NMR (400.1 MHz, CDCl_3_): *δ*=7.36–7.19 (m, 5H), 6.61 (s, 1H), 5.17 (s, 1H), 4.60 (s, 1H), 4.47–4.36 (m, 2H), 4.07 (s, 1H), 3.08 (t, *J*=6.7 Hz, 2H), 1.93–1.79 (m, 1H), 1.72–1.57 (m, 1H), 1.53–1.45 (m, 2H), 1.41 (overlap 2 s, 18H), 1.39–1.30 (m, 2H) ppm; ^13^C NMR (100.6 MHz, CDCl_3_): *δ*=172.2, 156.3, 156.0, 138.2, 128.8, 127.8, 127.6, 80.3, 79.4, 54.7, 43.6, 40.1, 32.0, 29.9, 28.6, 28.6, 28.4, 22.8 ppm; HRMS(ESI): *m/z* calcd for C_23_H_37_N_3_O_5_Na^+^: 458.2625, found 458.2623 [*M*+Na^+^].

**(2*S*)‐*N*‐Benzyl‐2,6‐diaminohexanamide trifluoroacetic acid salt (17)**: To a solution of (2*S*)‐*N*‐benzyl‐2,6‐bis(*tert*‐butoxycarbonylamino)hexanamide (**16**; 1.00 g, 2.30 mmol) in CH_2_Cl_2_ (30 mL) trifluoroacetic acid (10 mL) was added dropwise at 0 °C. The mixture was stirred at room temperature for 3 h. The solvent was removed under reduced pressure, the residue was redissolved in CH_2_Cl_2_, and the solvent was removed under reduced pressure once again to obtain the title compound (**17**) as a pale yellow oil (0.97 g, 2.09 mmol, 91 %). ^1^H NMR (400.1 MHz, [D_6_]DMSO): *δ*=8.95 (t, *J*=5.9 Hz, 1H), 8.19 (d, *J*=5.3 Hz, 3H), 7.78 (s, 3H), 7.44–7.12 (m, 5H), 4.44–4.38 (m, 2H), 3.78 (q, *J*=6.0 Hz, 1H), 2.82–2.61 (m, 2H), 1.82–1.65 (m, 2H), 1.57–1.47 (m, 2H), 1.38–1.25 (m, 2H). ppm; ^13^C NMR (100.6 MHz, [D_6_]DMSO): *δ*=168.4, 138.5, 128.4, 127.5, 127.2, 52.1, 42.4, 38.5, 30.5, 26.54, 21.25. ppm; HRMS(ESI): *m/z* calcd for C_13_H_22_N_3_O^+^: 236.17595, found 236.1766 [*M*+H^+^].

**General procedure A: Synthesis of unsaturated diamines (18 and 19)**: Under a nitrogen atmosphere 2,2‐dimethylpent‐4‐enal^[5]^ (2.4 equiv) as well as the corresponding diamine as its HCl/TFA salt were dissolved in anhydrous CH_2_Cl_2_ (10 mL/mmol of diamine) and sodium triacetoxyborohydride (3 equiv) was added portion wise at 0 °C, followed by acetic acid (2.4 equiv). The mixture was stirred at room temperature for 16–18 h and was then quenched by the addition of 20 % NaOH solution. The phases were separated and the aqueous layer was extracted three times with dichloromethane. The combined organic extracts were washed with brine, followed by distilled water and dried over MgSO_4_. The solvent was removed under reduced pressure and the crude product was obtained, which was used in the next step without further purification.

**(2*R*)‐Methyl‐2,6‐bis(2,2‐dimethylpent‐4‐enylamino)hexanoate (18)**: Was prepared according to general procedure **A** from d‐lysine methylester dihydrochloride (**14**; 2.13 g, 9.14 mmol) and 2,2‐dimethyl‐4‐pentenal (2.46 g, 21.93 mmol) yielding the title compound (**18**) as a pale yellow oil (2.73 g, 7.74 mmol, 85 %). ^1^H NMR (400.1 MHz, CDCl_3_): *δ*=5.89–5.66 (m, 2H), 5.06–4.90 (m, 4H), 3.70 (s, 3H), 3.12 (t, *J*=6.8 Hz, 1H), 2.58 (t, *J*=7.0 Hz, 2H), 2.39 (d, *J*=11.4 Hz, 1H), 2.34 (s, 2H), 2.08 (d, *J*=11.4 Hz, 1H), 2.04–1.92 (m, 4H), 1.68–1.13 (m, 8H), 0.94‐ 0.79 (m, 12H) ppm; ^13^C NMR (100.6 MHz, CDCl_3_): *δ*=176.6, 135.7, 135.6, 116.9, 116.9, 62.6, 60.4, 58.4, 51.6, 50.8, 44.9, 44.5, 34.6, 34.4, 33.6, 29.8, 25.7, 25.5, 25.4, 23.8 ppm; HRMS(ESI): *m/z* calcd for C_21_H_41_N_2_O_2_^+^: 353.3163, found 353.3176 [*M*+H^+^].

**(2*S*)‐*N*‐Benzyl‐2,6‐bis(2,2‐dimethylpent‐4‐enylamino)hexanamide (19)**: Was prepared according to general procedure **A** from (2*S*)‐*N*‐benzyl‐2,6‐diaminohexanamide trifluoroacetic acid salt (**17**; 1.15 g, 2.48 mmol) and 2,2‐dimethyl‐4‐pentenal (0.67 g, 5.95 mmol) yielding the title compound (**19**) as a pale yellow oil (0.84 g, 1.96 mmol, 79 %). ^1^H NMR (400.1 MHz, CDCl_3_): *δ*=7.57 (t, *J*=6.0 Hz, 1H), 7.36–7.29 (m, 2H), 7.29–7.21 (m, 3H), 5.94–5.62 (m, 2H), 5.08–4.90 (m, 4H), 4.47 (dd, *J*=14.7, 6.3 Hz, 1H), 4.38 (dd, *J*=14.7, 5.6 Hz, 1H), 3.01 (dd, *J*=7.9, 4.9 Hz, 1H), 2.66 (t, *J*=7.5 Hz, 2H), 2.41 (s, 2H), 2.37 (d, *J*=11.4 Hz, 1H), 2.18 (d, *J*=11.4 Hz, 1H), 2.07–2.00 (m, 2H), 1.97–1.88 (m, 2H), 1.84–1.71 (m, 1H), 1.65–1.49 (m, 3H), 1.47–1.33 (m, 2H), 0.93 (s, 6H), 0.81 (s, 6H) ppm; ^13^C NMR (100.6 MHz, CDCl_3_): *δ*=174.5, 138.7, 135.5, 135.2, 128.8, 127.9, 127.5, 117.4, 117.2, 63.7, 59.8, 59.5, 50.4, 44.8, 44.8, 43.1, 34.3, 34.2, 33.8, 28.9, 25.6, 25.4, 23.9 ppm; HRMS(ESI): *m/z* calcd for C_27_H_46_N_3_O_2_^+^: 428.3635, found 428.3621 [*M*+H^+^].

**General procedure B: Synthesis of bis‐*N*‐chloroamines (20 and 21)**: Under nitrogen, the crude unsaturated diamine was dissolved in anhydrous dichloromethane (10 mL/mmol of diamine), and freshly recrystallized *N*‐chlorosuccinimide (2.2 equiv) was added portion wise at 0 °C. The mixture was stirred at 0 °C for 30 min and for an additional 2–3 h at room temperature. The solvent was removed under reduced pressure and the crude product was purified by flash column chromatography.

**(2*R*)‐Methyl‐2,6‐bis(*N*‐chloro‐*N*‐(2,2‐dimethylpent‐4‐enyl)amino)‐hexanoate (20)**: Was prepared according to general procedure **B** from (2*R*)‐methyl‐2,6‐bis(2,2‐dimethylpent‐4‐enylamino)hexanoate (**18**; 2.73 g, 7.74 mmol) and purified by flash column chromatography (pentane/TBME 9 : 1) yielding the title compound (**20**) as a pale yellow oil (1.84 g, 4.37 mmol, 57 %). ^1^H NMR (400.1 MHz, CDCl_3_): *δ*=5.87–5.73 (m, 2H), 5.08–4.96 (m, 4H), 3.75 (s, 3H), 3.49 (t, *J*=7.2 Hz, 1H), 3.13 (d, *J*=15.2 Hz, 1H), 2.97‐2.81 (m, 5H, H‐6), 2.05 (d, *J*=7.5, 1.3 Hz, 4H), 1.89–1.78 (m, 2H), 1.72–1.62 (m, 2H), 1.61–1.50 (m, 1H), 1.45–1.34 (m, 1H), 0.98–0.89 (m, 12H) ppm; ^13^C NMR (100.6 MHz, CDCl_3_): *δ*=171.5, 135.4, 135.3, 117.5, 117.4, 75.0, 73.4, 72.4, 66.6, 51.8, 45.0, 44.7, 36.0, 35.7, 30.5, 27.9, 25.9, 25.7, 25.5, 23.5 ppm; HRMS(ESI): *m/z* calcd for C_21_H_39_Cl_2_N_2_O_2_^+^: 421.2389, found 421.2380 [*M*+H^+^].

**(2*S*)‐*N′*‐Benzyl‐2,6‐bis(*N*‐chloro‐*N*‐(2,2‐dimethylpent‐4‐enyl)amino)hexanamide (21)**: Was prepared according to general procedure **B** from (2*S*)‐*N*‐Benzyl‐2,6‐bis(2,2‐dimethylpent‐4‐enylamino)hexanamide (**19**; 1.20 g, 2.81 mmol) and purified by flash column chromatography (pentane/TBME 4 : 1) yielding the title compound (**21**) as a pale yellow oil (0.41 g, 0.82 mmol, 29 %). ^1^H NMR (400.1 MHz, CDCl_3_): *δ*=7.42–7.27 (m, 5H), 6.88 (t, *J*=5.9 Hz, 1H), 5.91–5.68 (m, 2H), 5.10–4.90 (m, 4H), 4.60–4.39 (m, 2H), 3.41 (dd, *J*=7.3, 5.4 Hz, 1H), 3.03–2.86 (m, 4H), 2.84 (s, 2H), 2.04 (ddt, *J*=15.7, 7.5, 1.3 Hz, 5H), 1.88 (ddt, *J*=13.7, 9.8, 5.8 Hz, 1H), 1.76–1.64 (m, 2H), 1.63–1.45 (m, 2H), 0.99–0.88 (m, 12H) ppm; ^13^C NMR (100.6 MHz, CDCl_3_): *δ*=170.6, 138.3, 135.4, 134.9, 128.8, 127.8, 127.6, 117.7, 117.5, 75.4, 75.0, 72.0, 66.5, 45.0, 45.0, 43.5, 35.9, 35.7, 29.0, 28.2, 25.9, 25.8, 24.7 ppm; HRMS(ESI): *m/z* C_27_H_44_Cl_2_N_3_O^+^: 496.2856, found 496.2850 [*M*+H^+^].

**General procedure C: Synthesis of bis‐3‐chloropiperidines (6 and 8)**: Under a nitrogen atmosphere the appropriate bis‐*N*‐chloroamine was dissolved in anhydrous chloroform (10 mL/mmol of bis‐*N*‐chloroamine) and tetrabutylammonium iodide (10 mol %) was added. The mixture was heated to 60 °C (oil bath temperature) for 2 h and the solvent was removed under reduced pressure. The product was purified by flash column chromatography. The resulting bis‐3‐chloropiperidines were obtained as an inseparable mixture of diastereomers.

**(2*R*)‐Methyl‐2,6‐bis(5‐chloro‐3,3‐dimethylpiperidin‐1‐yl)hexanoate (6)**: Was prepared according to general procedure **C** from (2*R*)‐methyl‐2,6‐bis(*N*‐chloro‐*N*‐(2,2‐dimethylpent‐4‐enyl)amino)hexanoate (**20**; 1.84 g, 4.37 mmol) and purified by flash column chromatography (pentane/TBME 9 : 1) yielding the title compound (**6**) as a pale yellow oil (0.88 g, 2.09 mmol, 48 %). ^1^H NMR (400.1 MHz, CDCl_3_) mixture of isomers: *δ*=4.12–3.91 (m, 2H), 3.68 (2x s, total 3H), 3.22–3.08 (m, 3H), 2.51–2.17 (m, 6H), 1.97–1.89 (m, 3H), 1.73–1.60 (m, 3H), 1.50–1.37 (m, 3H, H‐8), 1.37–1.25 (m, 3H), 1.01 (2x s, total 6H), 0.90 (2x s, total 6H) ppm; ^13^C NMR (100.6 MHz, CDCl_3_, selected signals) major isomer: *δ*=172.8, 66.9, 64.9, 64.2, 62.5, 61.7, 57.7, 55.2, 54.6, 54.3, 51.2, 48.8, 33.8, 33.4, 29.6, 29.2, 26.6, 25.4, 24.0 ppm; HRMS(ESI): *m/z* C_21_H_39_Cl_2_N_2_O_2_^+^: 421.2392, found 421.2388 [*M*+H^+^].

**(*2S*)‐*N*‐Benzyl‐2,6‐bis(5‐chloro‐3,3‐dimethylpiperidin‐1‐yl)hexanamide (8)**: Was prepared according to general procedure **C** from (2*S*)‐*N′*‐benzyl‐2,6‐bis(*N*‐chloro‐*N*‐(2,2‐dimethylpent‐4‐enyl)amino)‐hexanamide (**21**; 0.70 g, 1.41 mmol) and purified by flash column chromatography (pentane/TBME 1 : 1) yielding the title compound (**8**) as a pale yellow oil (0.47 g, 0.95 mmol, 67 %). ^1^H NMR (400.1 MHz, CDCl_3_) ∼1 : 1 isomeric mixture of **8^a^** and **8^b^**: *δ*=7.35–7.25 (m, 10H: 5H **8^a^** and 5H **8^b^**), 7.08 (s, 1H **8^a^**), 6.93 (s, 1H **8^b^**), 4.51–4.35 (m, 4H: 2H **8^a^** and 2H **8^b^**), 4.11–3.88 (m, 4H: 2H **8^a^** and 2H **8^b^**), 3.16‐3.03 (m, 4H: 2H **8^a^** and 2H **8^b^**), 3.01 (dd, *J*=7.6, 5.0 Hz, 1H 5H **8^a^**), 2.88 (t, *J*=6.4 Hz, 1H **8^b^**), 2.46–2.21 (m, overlapping signals 9H: 4H **8^a^** and 4H **8^b^** and 1H **8^a^**), 2.15–2.08 (m, 1H **8^b^**), 2.07–1.97 (m, 2H: 1H **8^a^** and 1H **8^b^**), 1.95–1.85 (m, 6H: 3H **8^a^** and 3H **8^b^**), 1.77–1.59 (m, 6H: 3H **8^a^** and 3H **8^b^**), 1.51–1.28 (m, 12H: 6H **8^a^** and 6H **8^b^**), 1.02 (s, 6H **8^a^**), 0.91 (s, 6H **8^b^**), 0.87 (d, *J*=10.0 Hz, 6H **8^a^**), 0.82 (s, 6H **8^b^**) ppm; ^13^C NMR (100.6 MHz, CDCl_3_, selected signals) major isomer: *δ*=172.7, 138.6, 128.8, 128.1, 127.7, 69.2, 64.8, 62.5, 62.4, 57.7, 57.5, 54.5, 53.9, 48.5, 48.0, 43.5, 33.4, 33.2, 29.5, 28.5, 27.2, 25.4, 24.4 ppm; HRMS(ESI): *m/z* C_27_H_44_Cl_2_N_3_O^+^: 496.2856, found 496.2856 [*M*+H^+^].

## Conflict of interest

The authors declare no conflict of interest.

## Supporting information

As a service to our authors and readers, this journal provides supporting information supplied by the authors. Such materials are peer reviewed and may be re‐organized for online delivery, but are not copy‐edited or typeset. Technical support issues arising from supporting information (other than missing files) should be addressed to the authors.

Supplementary
