# Supplementary material for: Appended Aromatic Moieties in Flexible Bis‐3‐chloropiperidines Confer Tropism against Pancreatic Cancer Cells
Source: ChemMedChem. 2020 Dec 3;16(5):860–8. doi: 10.1002/cmdc.202000814 (PMC7984046; doi:10.1002/cmdc.202000814)

ChemMedChem

Supporting Information

Appended Aromatic Moieties in Flexible Bis-3-chloropiperidines Confer Tropism against Pancreatic Cancer Cells

Caterina Carraro, Tim Helbing, Alexander Francke, Ivonne Zuravka, Alice Sosic, Michele De Franco, Valentina Gandin, Barbara Gatto,* and D. Richard Göttlich*

Author Contributions

C.C. Investigation:Lead

Table of contents

Supporting tables **S2**

Copies of ^{1}H and ^{13}C NMR spectra of the B-CePs 6 and 8 **S3**

Copies of HRMS spectra B-CePs 6 and 8 **S8**

		Relative percentage of detected reaction species					
Compound	Time	U	N^+	OH	N^+/OH	N^+/C=C	2OH
**3**	0 h	57.6	25.3	10.4	2.2	3.9	0.6
	1 h	3.7	2.5	4.8	61.1	0.7	27.2
	5 h	8.7	5.5	2.1	3.5	0.8	79.4
	O.N.	5.0	4.7	1.0	3.8	0.5	85.0
**5**	0 h	78.2	15.7	0.4	0.2	5.3	0.2
	1 h	39.2	15.0	0.5	28.3	6.0	11.0
	5 h	1.4	3.1	0.1	7.9	0.2	87.3
	O.N.	1.2	0.9	0.0	2.7	0.2	95.0
**6**	0 h	76.2	15.8	2.1	0.7	5.0	0.2
	1 h	39.9	14.0	2.3	24.1	5.9	13.8
	5 h	1.5	2.9	3.6	7.7	0.2	84.1
	O.N.	1.5	1.0	1.1	3.0	0.2	93.2
**7**	0 h	88.8	8.8	0.1	0.1	2.2	0.0
	1 h	67.3	19.7	0.9	2.5	7.1	2.5
	5 h	62.2	20.1	0.5	2.0	6.9	8.3
	O.N.	40.0	10.0	1.1	3.3	4.9	40.7
**8**	0 h	78.4	13.5	4.0	0.6	3.2	0.3
	1 h	62.8	7.8	0.7	14.9	3.1	10.7
	5 h	0.5	0.1	0.5	3.8	0.3	94.8
	O.N.	0.6	0.1	0.9	2.2	0.4	95.8
**9**	0 h	80.4	14.5	0.1	0.0	4.9	0.1
	1 h	66.7	21.3	0.3	1.9	9.0	0.8
	5 h	63.6	22.8	0.1	1.3	9.1	3.1
	O.N.	57.1	16.6	0.4	1.8	7.3	16.8
**10**	0 h	81.0	13.5	0.1	0.1	5.0	0.3
	1 h	67.1	19.3	0.6	1.5	9.6	1.9
	5 h	65.1	19.2	0.3	1.0	9.6	4.8
	O.N.	63.6	10.7	0.2	1.1	5.2	19.2
**11**	0 h	83.1	10.8	3.4	0.3	2.3	0.1
	1 h	74.8	12.7	4.7	1.5	4.9	1.4
	5 h	75.1	12.8	2.1	1.6	5.0	3.4
	O.N.	70.3	9.5	1.5	2.6	4.6	11.5
**12**	0 h	85.6	11.3	0.4	0.1	2.5	0.1
	1 h	77.6	16.1	0.8	0.7	4.6	0.2
	5 h	75.1	18.2	0.8	0.5	4.6	0.8
	O.N.	70.7	15.2	0.5	0.6	4.1	8.9
**13**	0 h	70.5	16.0	6.4	1.4	3.2	2.5
	1 h	46.8	20.1	23.4	1.2	7.4	1.1
	5 h	43.2	22.1	20.9	1.9	7.7	4.2
	O.N.	45.8	22.5	11.5	2.1	7.6	10.5

Table S1. Hydroxylation of B-CePs. Time-followed formation of B-CePs reaction species at 37 °C in BPE buffer pH 7.4 were detected by ESI-MS. The table reports the relative percentages of reaction species graphed in Figure 2, resulting from the incubation of test B-CePs in BPE buffer pH 7.4 at 37 °C for 0 h, 1 h, 5 h and overnight (O.N.) detected by ESI-MS.

Copies of ^{1}H and ^{13}C NMR spectra of the B-CePs 6 and 8

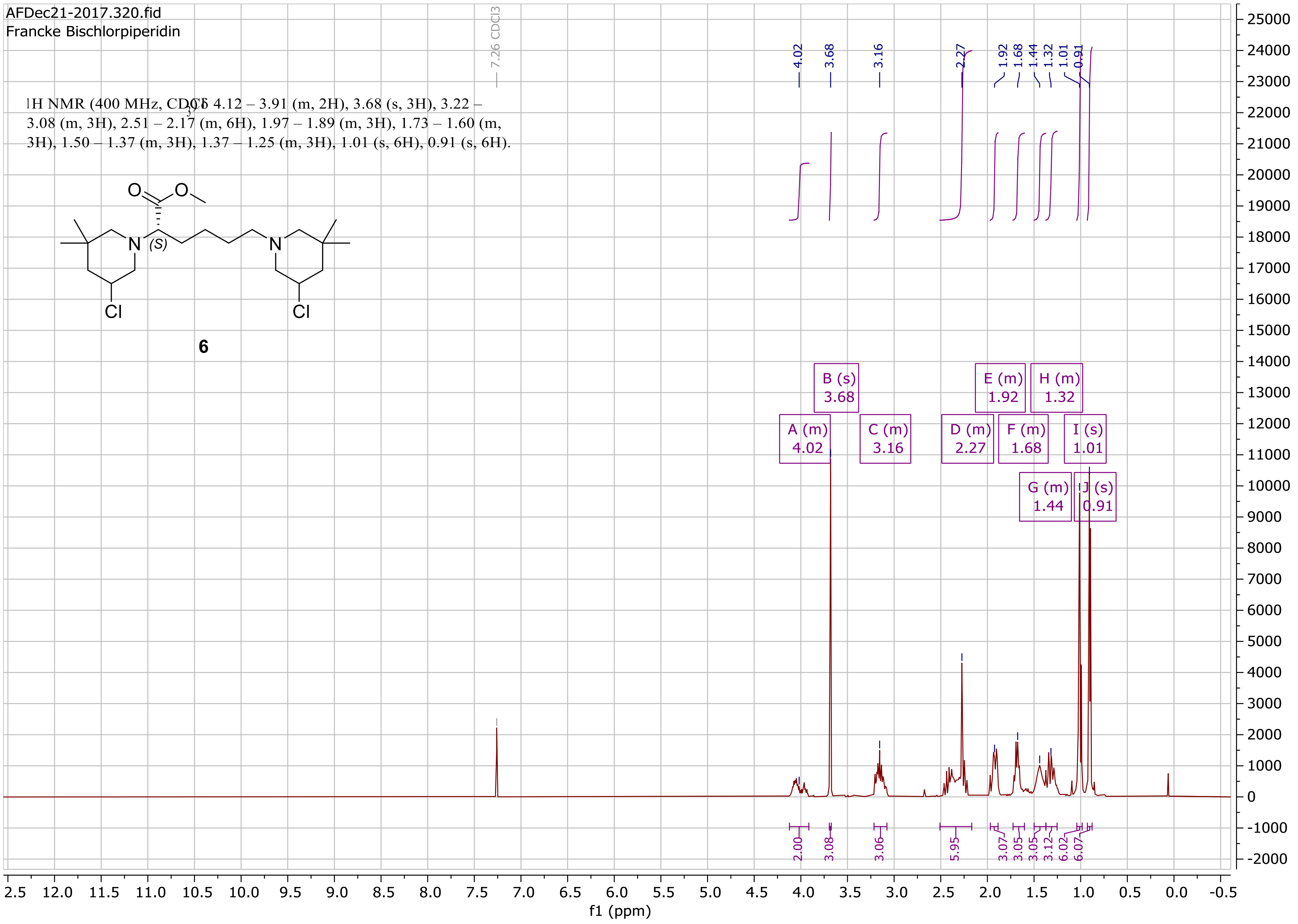
AFDec21-2017.320.fid
Francke Bischlorpiperidin
1H NMR (400 MHz, CDCl3) δ 4.12 – 3.91 (m, 2H), 3.68 (s, 3H), 3.22 – 3.08 (m, 3H), 2.51 – 2.17 (m, 6H), 1.97 – 1.89 (m, 3H), 1.73 – 1.60 (m, 3H), 1.50 – 1.37 (m, 3H), 1.37 – 1.25 (m, 3H), 1.01 (s, 6H), 0.91 (s, 6H).
6
7.26 CDCl3
A (m) 4.02
B (s) 3.68
C (m) 3.16
D (m) 2.27
E (m) 1.92
F (m) 1.68
G (m) 1.44
H (m) 1.32
I (s) 1.01
J (s) 0.91
f1 (ppm)

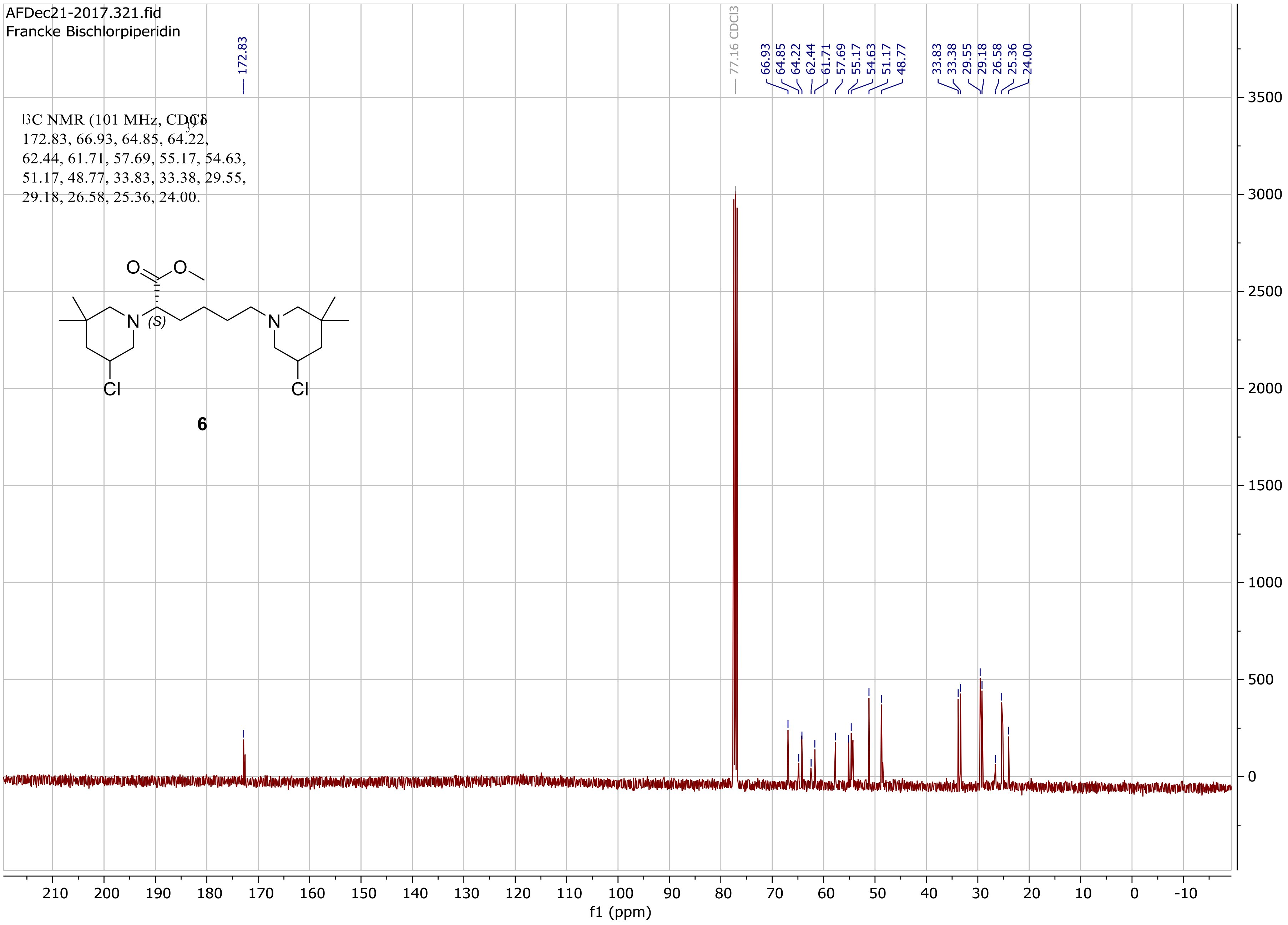

AFDec21-2017.321.fid
Francke Bischlorpiperidin
172.83
77.16 CDCl3
66.93
64.85
64.22
62.44
61.71
57.69
55.17
54.63
51.17
48.77
33.83
33.38
29.55
29.18
26.58
25.36
24.00
^{13}C NMR (101 MHz, CDCl$_3$) δ
172.83, 66.93, 64.85, 64.22,
62.44, 61.71, 57.69, 55.17, 54.63,
51.17, 48.77, 33.83, 33.38, 29.55,
29.18, 26.58, 25.36, 24.00.
O
O
N
(S)
N
Cl
Cl
6
3500
3000
2500
2000
1500
1000
500
0
210
200
190
180
170
160
150
140
130
120
110
100
90
80
70
60
50
40
30
20
10
0
-10
f1 (ppm)

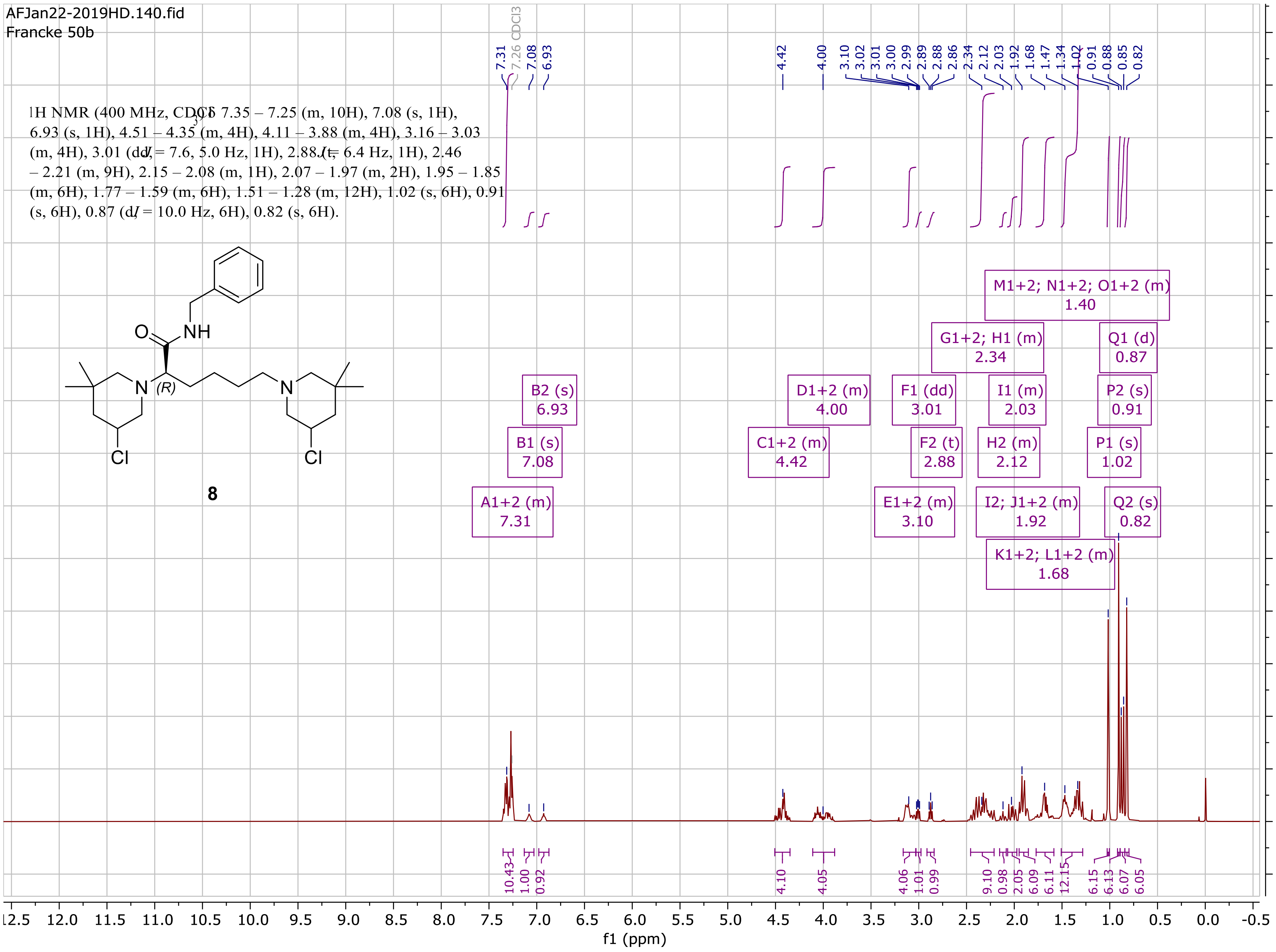
AFJan22-2019HD.140.fid
Francke 50b
1H NMR (400 MHz, CDCl3) δ 7.35 – 7.25 (m, 10H), 7.08 (s, 1H), 6.93 (s, 1H), 4.51 – 4.35 (m, 4H), 4.11 – 3.88 (m, 4H), 3.16 – 3.03 (m, 4H), 3.01 (dd, J = 7.6, 5.0 Hz, 1H), 2.88 (t, J = 6.4 Hz, 1H), 2.46 – 2.21 (m, 9H), 2.15 – 2.08 (m, 1H), 2.07 – 1.97 (m, 2H), 1.95 – 1.85 (m, 6H), 1.77 – 1.59 (m, 6H), 1.51 – 1.28 (m, 12H), 1.02 (s, 6H), 0.91 (s, 6H), 0.87 (d, J = 10.0 Hz, 6H), 0.82 (s, 6H).
O
NH
N
(R)
N
Cl
Cl
8
A1+2 (m) 7.31
B1 (s) 7.08
B2 (s) 6.93
C1+2 (m) 4.42
D1+2 (m) 4.00
E1+2 (m) 3.10
F1 (dd) 3.01
F2 (t) 2.88
G1+2; H1 (m) 2.34
H2 (m) 2.12
I1 (m) 2.03
I2; J1+2 (m) 1.92
K1+2; L1+2 (m) 1.68
M1+2; N1+2; O1+2 (m) 1.40
P1 (s) 1.02
P2 (s) 0.91
Q1 (d) 0.87
Q2 (s) 0.82
f1 (ppm)

AFJan22-2019HD.142.fid
Francke 50b

13C NMR (101 MHz, $CDCl_3$) δ 172.68, 138.56, 128.84, 128.09, 127.67, 69.24, 64.86, 64.76, 62.51, 62.44, 57.72, 57.50, 54.46, 53.87, 48.53, 48.04, 43.46, 33.38, 33.20, 29.54, 28.47, 27.22, 25.41, 24.41.

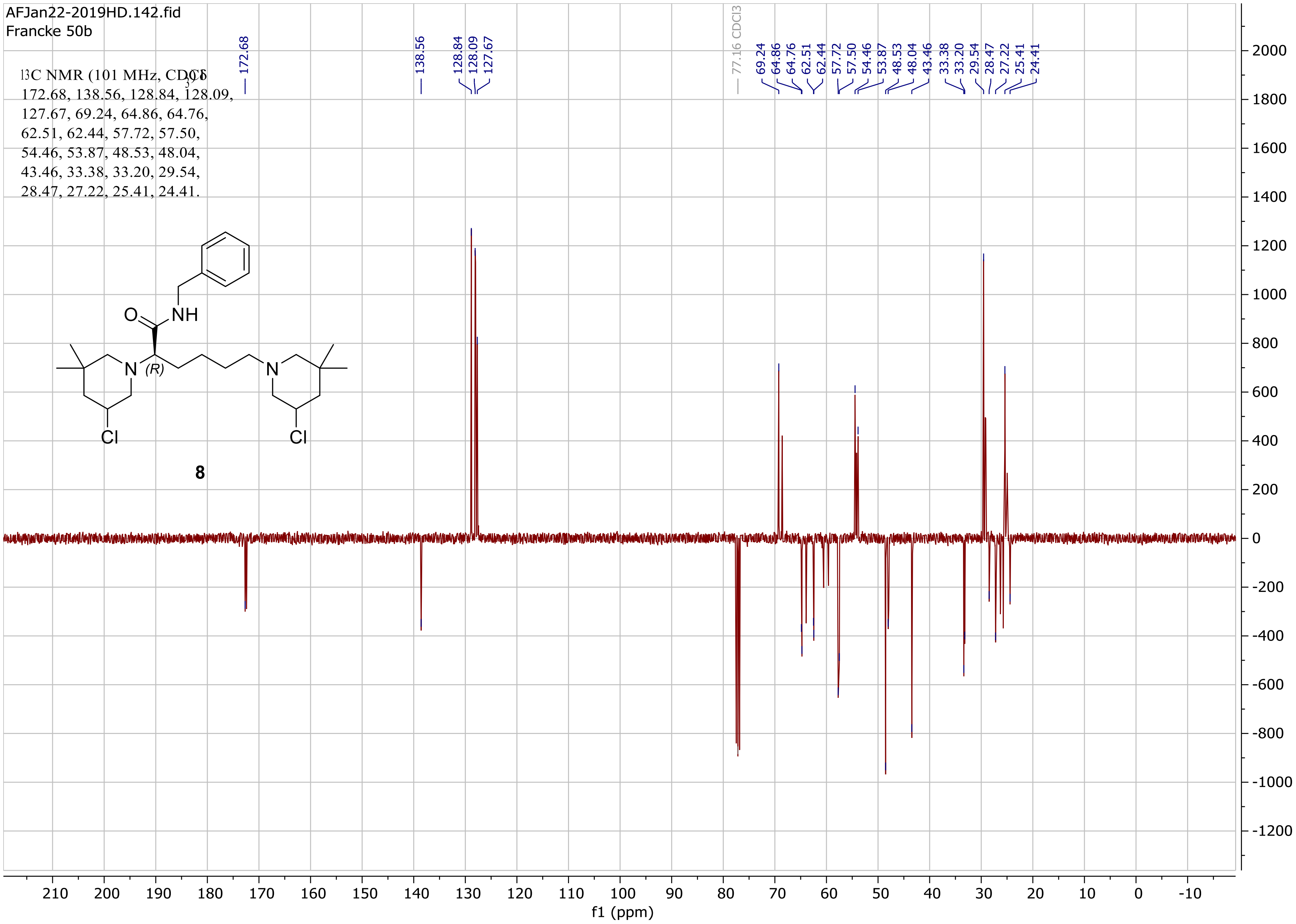

Copies of HRMS spectra of the B-CePs 6 and 8

Display Report

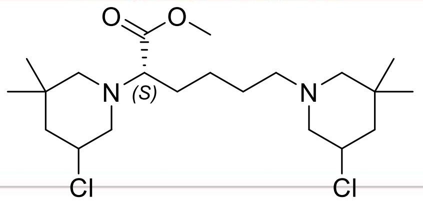

6

Analysis Info

Analysis Name	E:\AF000779.d
Method	tune_low_270308 pos.m
Sample Name	
Comment	

Acquisition Date	12/22/2017 1:55:42 PM	
Operator	finnigan-g	
Instrument / Ser#	micrOTOF	23

Acquisition Parameter

Source Type	ESI	Ion Polarity	Positive	Set Nebulizer	0.4 Bar
Focus	Not active			Set Dry Heater	180 °C
Scan Begin	50 m/z	Set Capillary	4500 V	Set Dry Gas	4.0 l/min
Scan End	1200 m/z	Set End Plate Offset	-500 V	Set Divert Valve	Waste

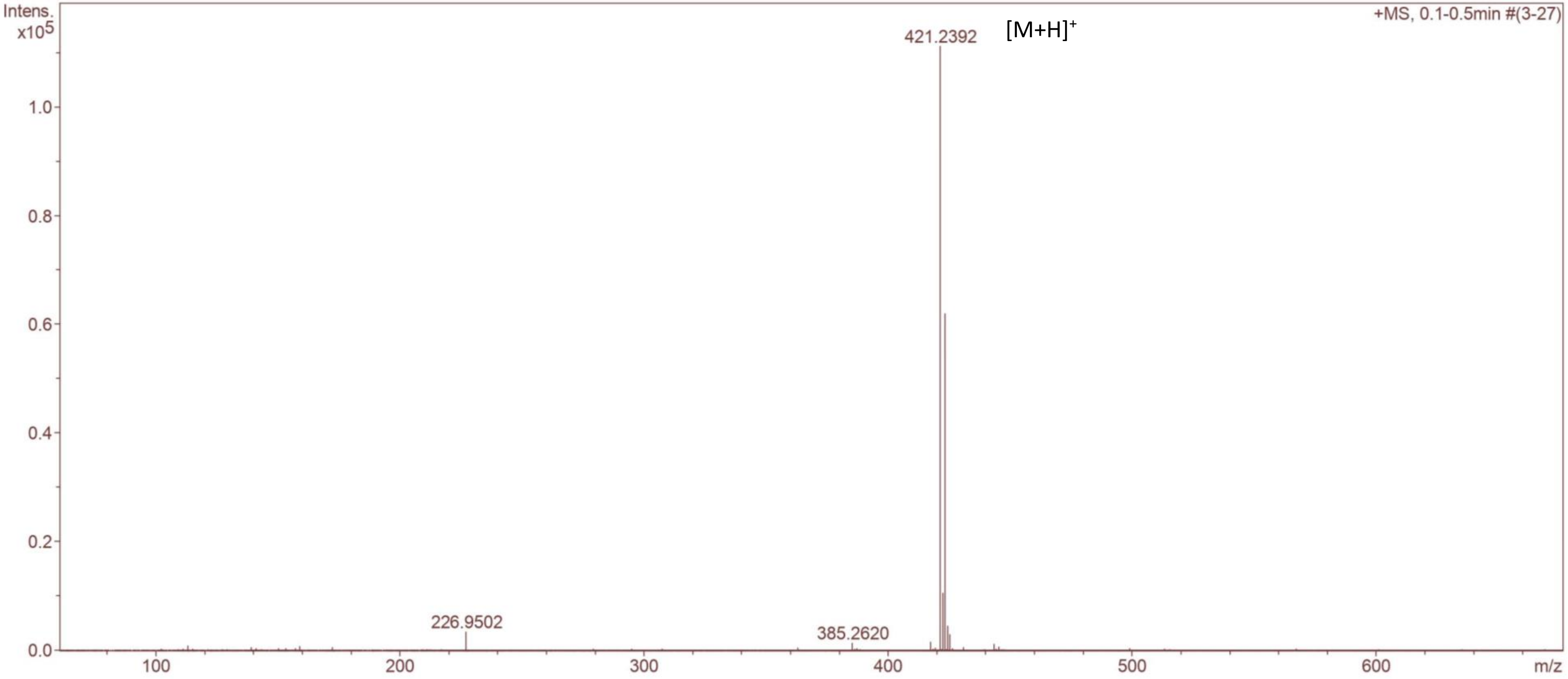

Display Report

Analysis Info

Analysis Name	E:\AF000987.d
Method	tune_low_270308 pos.m
Sample Name	50
Comment	DMSO/MeOH pos.

Acquisition Date	1/22/2019 11:17:28 AM
Operator	finnigan-g
Instrument / Ser#	micrOTOF 23

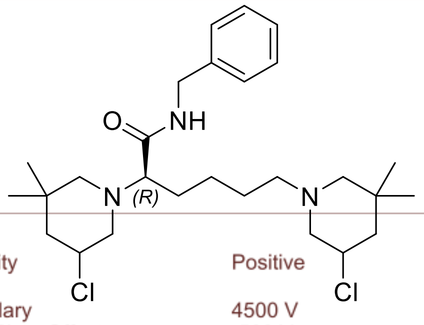

8

Acquisition Parameter

Source Type	ESI	Ion Polarity	Positive	Set Nebulizer	0.4 Bar
Focus	Not active			Set Dry Heater	180 ℃
Scan Begin	50 m/z	Set Capillary	4500 V	Set Dry Gas	4.0 l/min
Scan End	1400 m/z	Set End Plate Offset	-500 V	Set Divert Valve	Waste

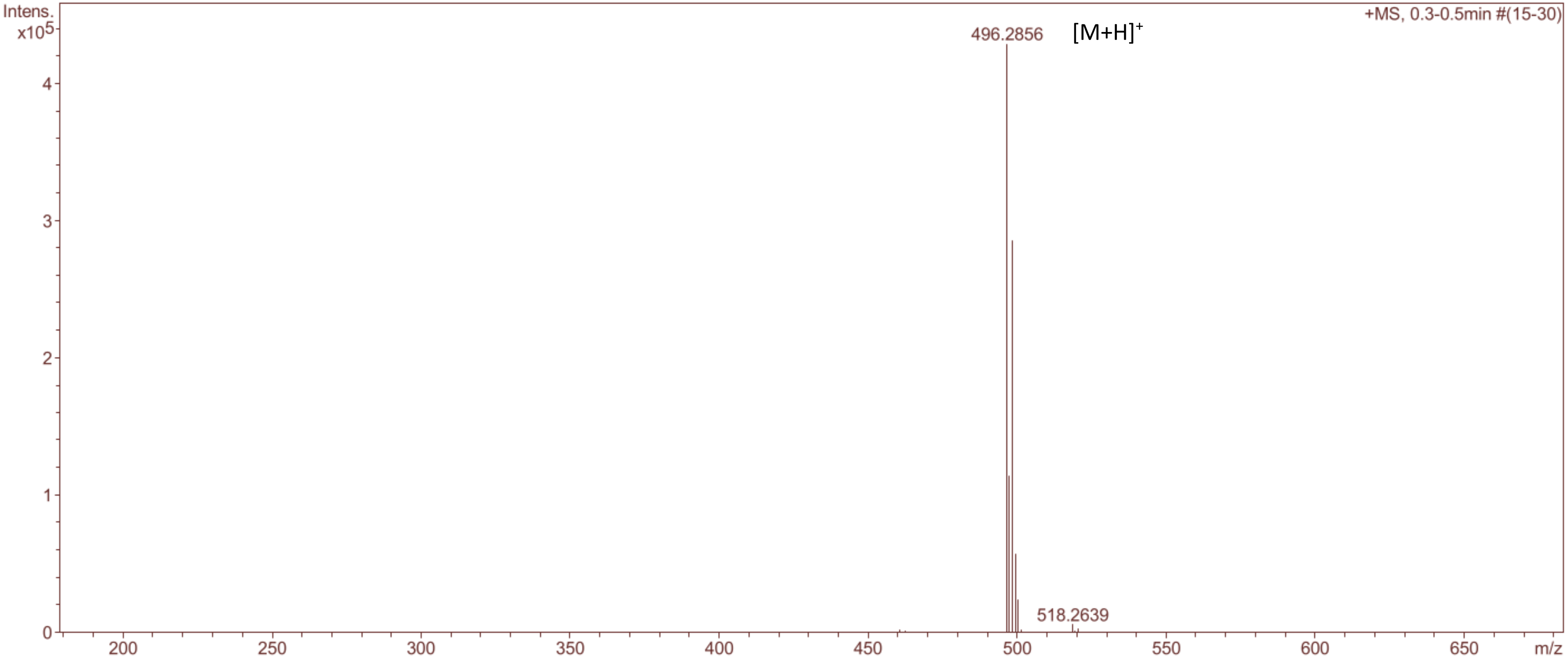

Supplement: Supplementary file 1 — Supplementary [file CMDC-16-860-s001.pdf]
